# Serum Antibodies to *Porphyromonas gingivalis* Chaperone HtpG Predict Health in Periodontitis Susceptible Patients

**DOI:** 10.1371/journal.pone.0001984

**Published:** 2008-04-23

**Authors:** Charles E. Shelburne, P. Sandra Shelburne, Vishnu M. Dhople, Domenica G. Sweier, William V. Giannobile, Janet S. Kinney, Wilson A. Coulter, Brian H. Mullally, Dennis E. Lopatin

**Affiliations:** 1 Department of Biologic and Materials Sciences, The University of Michigan School of Dentistry, Ann Arbor, Michigan, United States of America; 2 Department of Cariology, Restorative Sciences and Endodontics, The University of Michigan School of Dentistry, Ann Arbor, Michigan, United States of America; 3 Department of Periodontics and Oral Medicine and The Michigan Center for Oral Health Research, The University of Michigan School of Dentistry, Ann Arbor, Michigan, United States of America; 4 Centre for Oral Research, Queen's University, Belfast, Northern Ireland; The Research Institute for Children at Children's Hospital New Orleans, United States of America

## Abstract

**Background:**

Chaperones are ubiquitous conserved proteins critical in stabilization of new proteins, repair/removal of defective proteins and immunodominant antigens in innate and adaptive immunity. Periodontal disease is a chronic inflammatory infection associated with infection by *Porphyromonas gingivalis* that culminates in the destruction of the supporting structures of the teeth. We previously reported studies of serum antibodies reactive with the human chaperone Hsp90 in gingivitis, a reversible form of gingival disease confined to the oral soft tissues. In those studies, antibodies were at their highest levels in subjects with the best oral health. We hypothesized that antibodies to the HSP90 homologue of *P. gingivalis* (HtpG) might be associated with protection/resistance against destructive periodontitis.

**Methodology/Principal Findings:**

ELISA assays using cloned HtpG and peptide antigens confirmed gingivitis subjects colonized with *P. gingivalis* had higher serum levels of anti-HtpG and, concomitantly, lower levels of attachment loss. Additionally, serum antibody levels to *P. gingivalis* HtpG protein were higher in healthy subjects compared to patients with either chronic or aggressive periodontitis. We found a negative association between tooth attachment loss and anti-*P. gingivalis* HtpG (p = 0.043) but not anti-*Fusobacterium nucleatum* (an oral opportunistic commensal) HtpG levels. Furthermore, response to periodontal therapy was more successful in subjects having higher levels of anti-*P. gingivalis* HtpG before treatment (p = 0.018). There was no similar relationship to anti-*F. nucleatum* HtpG levels. Similar results were obtained when these experiments were repeated with a synthetic peptide of a region of *P. gingivalis* HtpG.

**Conclusions/Significance:**

Our results suggest: 1) anti-*P. gingivalis* HtpG antibodies are protective and therefore predict health periodontitis-susceptable patients; 2) may augment the host defence to periodontitis and 3) a unique peptide of *P. gingivalis* HtpG offers significant potential as an effective diagnostic target and vaccine candidate. These results are compatible with a novel immune control mechanism unrelated to direct binding of bacteria.

## Introduction

*Porphyromonas gingivalis* is a gram negative obligate anaerobe that has a major etiological role in human periodontitis. The bacterium is found with high frequency in persons with periodontitis where it participates in the initiation and establishment of chronic, infectious biofilms [1], [2]. These biofilms facilitate the long term survival of *P. gingivalis* and induces an inflammatory reaction that is responsible for the destruction of the hard and soft tissue supporting structures of the teeth. In addition *P. gingivalis* can invade and persist in the cells of the gingival tissue[3]. It also can escape the oral cavity and has been found in atherosclerotic plaque where it may have a role in the pathophysiology of cardiovascular disease [4].

*P. gingivalis* produces a number of chaperones as essential tools in normal cellular processes and in response to environmental stresses [5]. In addition, the role of chaperones, like the *P. gingivalis* HSP90 homologue HtpG, in immune response dynamics has become an area of intense investigation because immunomodulation by chaperones has been demonstrated [6], [7]. HtpG, like most chaperones tested [8], induces a strong humoral response that may have consequences in the pathogenesis of periodontitis [9]. These functions are important in the establishment and perpetuation of chronic inflammatory diseases.

Recent studies have shown that antibodies to periodontal disease associated pathogens may have protective effects although the exact mechanism is still unclear. Rams et al [10] demonstrated that serum levels of IgG antibodies against *A. actinomycetemcomitans* or *P. gingivalis* in periodontitis-stable patients were higher than those in patients with active periodontitis suggesting that these antibodies have a protective effect against periodontal infections. Yamasaki et al have shown that antibodies to *P. gingivalis* Hsp60 homologue increases with successful treatment [11]. We have recently described experiments that indicate that antibodies to HtpG may mitigate some of the induction of inflammatory chemokines through TLR4 and CD91 [12], a receptor expressed in human atherosclerotic lesions [13]. Taken together, these findings suggest a role for antibodies to *P. gingivalis* chaperones in both periodontal and cardiovascular disease.

The possible protective role of antibodies to chaperones in periodontitis is controversial. It has been suggested that these antibodies simply reflected the high level of homology between human and bacterial proteins, a hallmark of these evolutionarily conserved molecules [14]. Other results suggest that the conserved nature of the chaperones might lead to autoimmune phenomenon due to “immune mimicry”. Earlier results from this laboratory suggested that high levels of anti-Hsp90 antibodies could have protective qualities [9]. However, that study utilized a group of individuals with minimal periodontal disease. Here we describe findings from a study of periodontitis subjects with more extensive disease that are similar to those reported earlier and support the notion of a protective role for anti-HtpG antibodies in untreated subjects. In addition, these studies suggest that the levels of these antibodies also may predict the response to treatment. These results may also reflect an immune control mechanism in chronic infections unrelated to direct binding of bacteria.

## Results

### Colonization of plaque by *P. gingivalis* and *F. nucleatum*

Initially we reported [9] that in gingivitis subjects probing depth and the levels of *P. gingivalis* were both associated with serum anti-Hsp90 antibody levels. Subjects from the gingivitis group were tested for the presence of *P. gingivalis* by slot immunoblot; 82 % of the subjects were positive for *P. gingivalis* [15], [16]. Genomic DNA of pooled plaque samples from all teeth for each subject in the CP and AP groups were tested; 71% of the healthy subjects and 86% of individuals with periodontitis were positive for *P. gingivalis* 16S rRNA genes by PCR. The low end sensitivity of the assay was between 10 and 100 bacterial cells per sample. The mean percentage of *P. gingivalis* was 0.11 and 0.98 (p<0.001, *t*-test) in the controls and periodontitis subjects respectively. Both CP groups were 96% positive for *F. nucleatum*; the mean percentage of *F. nucleatum* was 2.77 and 3.78 (p = 0.07; *t*-test) in the controls and periodontitis subjects respectively (Table 1). Plaque samples from the normal controls for the AP group were not available for *F. nucleatum* testing and as reported previously 17% of the AP subjects were positive for *P. gingivalis* 16S rRNA [17].

**Table 1:** Research Subject Demographics

*Group (n)*	*Gingvitis (50)*	*CP-Healthy (49)*	*CP-Diseased (50)*	*AP-Healthy (21)*	*AP-Diseased (24)*
% Female	58	54	58	62	68
Age Range in years (mean)	18-66 (31.6)	20-78(43.0)	30-77(51.3)	18-35 (34.4)	22-40(32.0)
Attachment Loss (mm, mean±SD)	1.56±0.70	0.68±0.52	2.24±1.07	0.72±0.52	3.25±0.63
Probing Depth (mm, mean±SD)	2.64±0.54	1.60±0.21	2.60±0.71	1.50±0.22	2.83±1.32
Bleeding on Probing (%, mean±SD)	ND	0.24±0.12	0.57±0.17	0.13±0.06	0.33±0.16
Gingival Index (%, mean±SD )	0.2±0.17	0.18±0.19	0.52±0.23	ND	ND

ND–not determined

### Antibodies to *P. gingivalis* and *F. nucleatum* total cell lysates

The level of antibodies to total cell lysates of *P. gingivalis* and *F. nucleatum* was determined as a measure of the long term colonization experience that each subject had with these bacteria. These two bacteria have different roles in periodontitis, *P. gingivalis* as a pathogen while *F. nucleatum* is an opportunistic commensal in the subgingival biofilm. Anti-*P. gingivalis* antibodies were detected in 38 of 45 samples obtained from gingivitis subjects. All CP and AP subjects had levels of anti-*P. gingivalis* and anti-*F. nucleatum* more than 3 standard deviations above the background of the assay. In the CP group there was a statistically significant elevated levels of antibodies to *P. gingivalis* (p = 0.01, *t*-test) and *F. nucleatum* (p = 0.02, *t*-test) in the diseased subjects compared to the control group; there was no difference between levels of antibody to total *P. gingivalis* in the AP group (Table 2). Similar results were found in CP subjects that were colonized by *P. gingivalis* (Table 3) and CP subjects not colonized by *P. gingivalis* (Table 4) at the time the serum samples were obtained. These results suggest that most subjects in the study were likely to have been colonized with *P. gingivalis* prior to their participation in this study.

**Table 2:** Antibody responses to *P. gingivalis* and *F. nucleatum* in subjects.

*Group (n)*	*Gingivitis (50)*	*CP-Healthy (49)*	*CP-Diseased (50)*	*AP-Healthy (21)*	*AP-Diseased (24)*
% positive for *P. gingivalis*	82	71	86	ND	17
% positive for *F. nucleatum*	ND	96	96	ND	ND
Antibody to total *P. gingivalis* (Log mean ELISA Units±S.D.)	4.39±0.17	3.68±0.23	3.92±0.26	4.47±0 .07	4.39±0.15
Antibody to total *F. nucleatum* (Log mean ELISA Units±S.D.)	ND	3.95±0.62	4.86±0.81	ND	ND

ND–not determined

**Table 3:** Serum antibody (IgGγ) levels to bacterial antigens in CP subjects colonized by *P. gingivalis*.

	*ELISA Units*			
	Health (mean)	Disease (mean)	p-value (*t*-test)	Valid N (Health/Disease)
Anti-*P.gingivalis* HtpG	10,200	9,882	NS	35/41
Anti-*F.nucleatum* HtpG	19,959	19,817	NS	35/38
Anti-*P.gingivalis* HtpG p18	7,138	6,254	0.047	35/40
Anti-*P.gingivalis* W83	5,654	10,076	0.002	35/40
Anti-*F.nucleatum* 22586	27,735	13,701	0.009	35/41

NS-not significant

**Table 4:** Serum antibody (IgGγ) levels to bacterial antigens in CP subjects not colonized by *P. gingivalis*.

	*ELISA Units*		p-value (*t-*test)	Valid N (Health/Disease)
	Health (mean)	Disease (mean)		
Anti-*P.gingivalis* HtpG	9,224	8,594	NS	13/6
Anti-*F.nucleatum* HtpG	19,818	15876	NS	13/7
Anti-*P.gingivalis* HtpG p18	6,812	6,477	NS	13/7
Anti-*P.gingivalis* W83	5,347	11,008	0.002	13/7
Anti-*F.nucleatum* 22586	17,917	8,473	0.009	13/7

NS-not significant

### Total IgG Antibodies to *P. gingivalis* HtpG and *F. nucleatum* HtpG

Levels of IgG-class antibodies that specifically reacted with recombinant HtpG proteins of *P. gingivalis* and *F. nucleatum were* measured. Anti-HtpG antibody levels were measured in gingivitis subjects; subjects with more CAL (more tissue loss) had lower levels of anti-*P. gingivalis* HtpG.

In the CP subjects we hypothesized that the pattern of anti-HtpG would be different for the two bacteria. However, there was a trend, both in CP individuals colonized (Table 3) or un-colonized (Table 4) by *P. gingivalis,* to higher antibody levels to HtpG from both species in healthy subjects than in subjects with disease even though this trend did not reach statistical significance.

There were substantial and significant differences between the healthy and diseased AP subjects in all the variables tested. The three clinical measurements were all significantly higher in the diseased groups (Table 5) while the anti-HtpG levels were significantly lower in the diseased subjects (p<0.002, ANOVA).

**Table 5:** Serum antibody (IgGγ) levels to bacterial antigens in AP subjects.

	*Health (mean±S.D)*	*Disease (mean±S.D.)*	*p-value (ANOVA)*	*Valid N*
PD (mm)	1.51±0.22	3.31±0.67	<0.002	45
AL (mm)	0.719±0.52	2.85±1.45	<0.002	45
BOP (%)	0.125±0.07	0.29±0.17	<0.002	45
Log anti-p18	4.11±0.27	3.97±0.16	0.041	45
Log anti-HtpG	4.25±0.04	3.96±0.13	<0.002	45
Log anti-W83 cells	4.47±0.07	4.39±0.15	0.032	45

### Antibodies to *P. gingivalis* HtpG peptide p18

Previous studies [18] suggested that a peptide antigen, p18, of the HtpG molecule was responsible for eliciting the apparent protective effect found in the serum of healthy subjects. Therefore the serum response to a synthetic peptide antigen of this region was investigated in the CP and AP subjects. We expected that the levels of antibody to the peptide would be higher in control subjects compared to periodontitis subjects of both groups and that elimination of the majority of the epitopes in the HtpG molecules, most of which are highly conserved between bacterial species, would clarify the trend observed using the whole HtpG proteins as ELISA target antigens. As expected, subjects in both the CP (Table 3 & 4) and AP (Table 5) groups diagnosed with periodontitis had lower levels of anti-p18 than their respective control groups. Although the same trend was observed in both colonized and un-colonized individuals using the HtpG molecule only the differences in p18 antibody levels in CP subjects colonized by *P. gingivalis* reached statistical significance (Table 3 & 4).

### Cluster Analysis by Disease Subgroups

There were trends supporting our hypothesis when the results of the assays were compared between groups as they were recruited into the studies, basically health or disease status. However, examination of those groupings showed that there was overlap between the groups based on clinical measures, especially in the CP subjects, the most complete study. Therefore the subjects were regrouped in line with their clinical measures by K-means clustering, irrespective of recruitment groups. The clusters were used as a basis for ANOVA analysis of differences in the clinical measures and antibody levels.

Gingivitis subjects were regrouped into three clusters and differences in four clinical measures (PD, CAL, GI, PL) and two antibody levels (anti*-P. gingivalis* HtpG, anti-P. gingivalis whole cell lysate) sought. Cluster 1 had the greatest PD and CAL; there were significant differences between the clusters for PD and CAL. Antibody levels against *P. gingivalis* HtpG were lowest in the subjects with the highest disease measures, similar to earlier findings [9] for human Hsp90, but did not reach statistical significance (Table 6).

**Table 6:** Analysis of clustered subjects in the Gingivitis group.

	*Cluster 1*	*Cluster 2*	*Cluster 3*	*p*-value (ANOVA)
	Mild perio. n = 22	Gingivitis n = 10	Healthy n = 18	
PD	3.1±0.2	2.8±0.4	2.05±0.2	<0.001
AL	2.2±0.6	1.0±0.0	1.11±0.3	<0.001
GI	27.3±5.5	30.0±4.8	5.5±2.7	NS
Pl	45.5±5.9	8.0±6.3	27.8±4.6	NS
Anti*-P. gingivalis* HtpG	9825±4720	14973±10178	12676±6928	NS
Anti-P. gingivalis W83	3100±6898	1301±177	7488±11008	NS

NS–not significant

Subjects were clustered by K-means clustering and differences sought in means (±S.D.) of variables by ANOVA. PD and AL measured in mm; GI is the % sites with gingival redness; PI values are % sites with accumulated plaque. Antibody values are expressed in ELISA units.

CP Subjects were regrouped into 4 clusters and differences sought in four clinical measures (PD, CAL, BOP, GI) and three antibody levels (anti*-P. gingivalis* HtpG, anti-Fn HtpG and anti-Pg p18). There were significant differences between the groups in all clinical measures (Table 7). Cluster 2 had the highest levels of all clinical measures and the lowest levels of anti*-P. gingivalis* HtpG and anti*-P. gingivalis* HtpG p18, the latter being significant (p = 0.00056, ANOVA).

**Table 7:** Cluster analysis of CP subjects.

	*Cluster 1*	*Cluster 2*	*Cluster 3*	*Cluster 4*	*p*-value (ANOVA)
	Moderate CP n = 31	Severe CP n = 10	Gingivitis n = 29	Healthy n = 27	
PD	2.4±0.4	3.6±0.8	1.7±0.2	1.6±0.2	<0.001
AL	1.9±0.1	4.0±0.9	1.2±0.3	0.3±0.2	<0.001
BOP	56.8±16.2	68.4±16.6	27.7±.12.7	24.6±14.6	<0.001
GI	54.3±21.1	60.6±24.6	20.2±17.9	21.4±21.0	<0.001
Anti*-P. gingivalis* HtpG	10531±4791	7877±3906	9311±3833	10358±4443	NS
Anti-F. *nucleatum* HtpG	20053±806	18530±9346	18231±5752	20748±6540	NS
Anti-*P. gingivalis* HtpG p18	6485±1920	4793±2120	7171±1782	6957±1068	0.006

NS–not significant

Subjects were clustered by K-means clustering and differences sought in means (±S.D.) of variables by ANOVA. PD and AL measured in mm; GI is the % sites with gingival redness. Antibody values are expressed in ELISA units.

AP subjects were regrouped into 3 clusters and differences sought in three clinical measures (PD, CAL, BOP) and three antibody levels (anti-Pg whole bacteria, anti*-P. gingivalis* HtpG and anti-Pg P18). There were significant differences in all the clinical measures between groups and in anti*-P. gingivalis* HtpG and anti*-P. gingivalis* HtpG p18, but not anti-Pg whole bacteria (Table 8). Cluster 2 had the highest levels of clinical measures and lowest levels of anti*-P. gingivalis* HtpG p18. Both clusters 1 and 2 had substantially lower levels of anti*-P. gingivalis* HtpG compared to cluster 3.

**Table 8:** Cluster analysis of AP subjects.

	*Cluster 1*	*Cluster 2*	*Cluster 3*	*p-value (ANOVA)*
	Localized AP n = 11	Generalized AP n = 12	Healthy n = 21	
PD	3.0±0.6	3.6±0.7	1.5±0.2	<0.001
AL	2.2±1.2	3.4±1.5	0.7±0.5	<0.001
BOP	21.4±11.2	37.9±18.1	12.5±5.7	<0.001
Anti-P. gingivalis HtpG p18	10182±2437	8371±2114	12390±5318	0.0283
Anti*-P. gingivalis* HtpG	8853±2493	10165±3479	17742±1349	<0.001
Anti-P. gingivalis W83	26899±9509	26040±12191	30041±4977	NS

NS–not significant

Subjects were clustered by K-means clustering and differences sought in means (±S.D.) of variables by ANOVA. PD and AL measured in mm; BOP is the % sites bleeding on probing. Antibody values are expressed in ELISA units. NS–not significant.

### Correlation of Anti-*P. gingivalis* HtpG antibodies to clinical measures in Chronic and Aggressive Periodontitis

The supposition that antibodies to *P. gingivalis* HtpG may be protective is based on the hypothesis that high levels of antibodies should be found in subjects exhibiting healthier clinical signs and lower levels in subjects exhibiting more periodontitis related damage. Clinical measurements from each site in all the subjects were obtained and the average value determined for each subject. Relationships between those measurements and the levels of *P. gingivalis* and *F. nucleatum* anti-HtpG were sought using Pearson's R to compare baseline antibody levels in both AP and CP groups with the clinical signs obtained at the baseline were compared. When anti-*P. gingivalis* HtpG levels from CP subjects were analyzed with four clinical measurements in each of the same patients there was a trend for pocket depth and attachment loss measurements to inversely correlate with antibody levels, but the correlations were small and not statistically significant. Similar results were found with anti-*F. nucleatum* HtpG values. However, in the AP subjects there were substantial negative associations three clinical measurements and anti-HtpG levels, all of which were statistically significant (Table 9). Similar experiments using HtpG cloned from *F. nucleatum* gave no significant correlations to the clinical signs (data not shown).

**Table 9:** Correlation of Anti*-P. gingivalis* HtpG antibody levels with clinical measurements.

		*A. Pretreatment*				*B. Post-treatment*			
		Anti-*P. gingivalis* HtpG		Anti-*F. nucleatum* HtpG		Anti-*P. gingivalis* HtpG		Anti-*F. nucleatum* HtpG	
		Pearson's R	*p*-value	Pearson's R	*p*-value	Pearson's R	*p*-value	Pearson's R	*p*-value
Gingivitis									
	PD	−0.111	0.440						
	AL	−0.341	0.012						
	GI	−0.101	0.483						
	PI	−0.130	0.367						
CP									
	PD	−0.050	NS	−0.019	NS	0.361	0.002	0.076	NS
	AL	−0.108	NS	−0.113	NS	0.273	0.020	0.144	NS
	BOP	0.144	NS	0.057	NS	0.381	0.001	0.113	NS
	GI	0.093	NS	0.001	NS	0.263	0.026	0.140	NS
AP									
	PD	−0.787	<0.001	ND					
	AL	−0.628	<0.001	ND					
	BOP	−0.388	<0.01	ND					

NS–not significant

ND–not determined

### Correlation of Anti-*P. gingivalis* HtpG p18 antibodies to clinical measures in Chronic and Aggressive Periodontitis

We then sought relationships between the same measurements and the levels of *P. gingivalis* anti-HtpG p18 by comparing antibody levels in both AP and CP groups with the clinical measurement using Pearson's R analysis. Sera from the CP group and controls showed that levels of anti-p18 were significantly (p = 0.047) inversely correlated to CAL but not correlated to the other indices. In the AP group antibodies to p18 were significantly inversely correlated to PD (p = 0.008), CAL (p = 0.018) and BOP (p = 0.046), Table 10. Lastly, associations were sought between changes in clinical measurements 6 months after SRP treatment and the anti-p18 levels 6 months before treatment to determine if they might predict treatment success or failure. There was a significant positive correlation (p = 0.05) between the pre-treatment levels of anti-HtpG p18 and reduction in CAL.

**Table 10:** Correlation of Anti*-P. gingivalis* HtpG p18 antibody levels with clinical measurements.

		*Pre-treatment*		*Post-treatment*	
		Anti-*P. gingivalis* HtpG *p18*		Anti-*P. gingivalis* HtpG *p18*	
		Pearson's R	*p*-value	Pearson's R	*p-*value
Gingivitis					
	PD	−0.135	NS		
	AL	0.066	NS		
	GI	−0.081	NS		
	PI	0.136	NS		
CP					
	PD	−0.063	NS	0.072	NS
	AL	−0.204	0.047	0.153	NS
	BOP	−0.100	NS	−0.119	NS
	GI	−0.111	NS	−0.084	NS
AP					
	PD	−0.393	0.008		
	AL	−0.361	0.015		
	BOP	−0.298	0.046		

NS–not significant

## Discussion

Chaperones are simultaneously highly conserved and immunodominant antigens which may have important roles in the pathogenesis of numerous human diseases. Although a subject of intense investigation, the role of the immune response to chaperones in human disease is currently not well understood. Periodontal diseases present a unique opportunity to examine the immune response to chaperones in a distinctive and readily accessible human environment. A few reports of antibody levels to *P. gingivalis* chaperones (particularly GroEL) have been published but this is the first comprehensive analysis of anti-*P. gingivalis* HtpG in multiple periodontal disease states. Results reported here and earlier [9], [12] suggest that anti-*P. gingivalis* HtpG antibodies predict health in patients susceptible to periodontal disease and are protective in the untreated periodontal disease patient. These results may also reflect a previously uncharacterized immune control mechanism unrelated to direct binding of bacteria. Further, these antibodies may augment periodontitis treatment and *P. gingivalis* HtpG might be an attractive vaccine candidate. These results appear to be unique to *P. gingivalis* as they are directed at a segment of HtpG unique to *P. gingivalis*; parallel experiments with the HtpG homologue of *F. nucleatum* did not manifest these same qualities. In addition, there is reason to believe that these results may be extended to other chronic infectious diseases.

Earlier studies of a population of subjects with minimal periodontal disease suggested that antibodies to human Hsp90 related chaperones might have a protective effect. This report expands on that notion by examining the response of periodontitis subjects to the Hsp90 homologue in *P. gingivalis*, HtpG. In gingivitis subjects as a whole it was found that there was a distinctive, but not statistically significant, trend in support of the hypothesis. Interestingly, when anti*-P. gingivalis* HtpG levels and clinical measures are compared on an individual basis there is a discernable correlation with CAL. At the opposite end of the periodontitis disease spectrum, the AP subjects, there is considerable support for the hypothesis. At the group level, there are substantial and significant differences between both anti*-P. gingivalis* HtpG and anti*-P. gingivalis* HtpG p18 between clusters based on clinical measures, but no differences in the response to the whole bacterium. On an individual basis, both anti*-P. gingivalis* HtpG and anti*-P. gingivalis* HtpG p18 are inversely and significantly correlated to the clinical measures.

Support of the hypothesis was somewhat tenuous in the initial examination of the CP subjects. Those recruited as either healthy or with CP and colonized by *P. gingivalis* possessed a humoral response to *Pg* HtpG p18 higher in subject groups with less periodontal destruction and inversely related to the level of attachment loss (p<0.05). A similar relationship was not found in un-colonized individuals. This was the opposite of antibody levels to the whole bacterium which were significantly higher is subjects with disease than controls (p≤0.01), a finding reported by many other laboratories. Similar trends were found when we examined the humoral response to HtpG from *F. nucleatum* but there was no relationship to disease status. In addition, correlations between the same antibodies and clinical measures when considered in the context of individual subjects were not found, except for a correlation between ant-*P. gingivalis* HtpG p18 and CAL (R = −0.205, p<0.05). This dilemma was resolved when the subjects were clustered by clinical measures. In a cluster of 10 subjects with the most severe disease there was a substantial and significantly lower level of anti*-P. gingivalis* HtpG and anti*-P. gingivalis* HtpG p18 compared to the other clusters in the CP subjects. As might be expected, the clinical measures of these subjects resemble those of the AP subjects. In summary, there is a trend for both anti*-P. gingivalis* HtpG and anti-*P. gingivalis* HtpG p18 to be lower in subjects with the more serious disease in each of the 3 periodontal disease groups. Neither trend reaches statistical significance in the gingivitis group. In the CP group anti-*P. gingivalis* HtpG p18 is significantly lower in the most diseased cluster; anti*-P. gingivalis* HtpG trends in the same direction but does not reach statistical significance. In the AP group both anti*-P. gingivalis* HtpG and anti-*P. gingivalis* HtpG p18 are significantly lower in the diseased clusters of subjects. Our initial observations (16) were probably due to the response of the most diseased individuals in that group of subjects. However, we believe that the current results are not fortuitous because 1) the relationship between the antibody levels and disease is evident not only in the original group but in groups with more serious disease in an almost dose dependent manner; 2) there is a relationship between these antibody levels and response to periodontal treatment; 3) there is a link through these antibodies to cellular receptors involved in antigen recognition and inflammation; 4) similar results have been noted in other chronic bacterial infections. In addition, there is no evidence that the lower levels of anti-*P. gingivalis* HtpG are due to adsorption by high circulating levels of either human Hsp90. There was no significant correlation between individual serum levels of human Hsp90 protein and anti-*P. gingivalis* HtpG, anti-*F. nucleatum* HtpG or anti-*P. gingivalis* HtpG p18 (Table S1). In fact, there was a trend to higher levels of anti-*P. gingivalis* HtpG in subjects with higher levels of human Hsp90, the opposite of what be expected if such absorption was taking place. There was also no significant difference between levels of human Hsp90 in healthy subjects with high levels of anti-*P. gingivalis* HtpG antibodies and severe periodontitis subjects with low levels of anti-*P. gingivalis* HtpG (Table S2). Lastly, while it is possible that a subset of anti-*P. gingivalis* HtpG antibodies might bind other HSP90 homologues, including human Hsp90, the *P. gingivalis* HtpG p18 epitope is unique and antibodies to that peptide could not be adsorbed by other HSP90 family proteins (see discussion below).

Subjects with CP who were given periodontal treatment responded more effectively to that treatment the higher their original pretreatment levels of anti-*P. gingivalis* HtpG. There was no similar effect with antibodies to *F. nucleatum* HtpG. Notably, the serum samples, which are time-averaged values, correlate best with clinical indices related to tissue destruction and less with indices related to inflammation. This suggests that these anti-bodies are found in subjects predisposed to a better treatment outcome or they may somehow facilitate healing after treatment. Interestingly, drugs that inhibit Hsp90 have been shown to prolong survival, attenuate inflammation, and reduce lung tissue injury in murine sepsis [19].

These findings are also worthy of note because despite high homology among chaperones in diverse organisms, the *P. gingivalis* HtpG is dissimilar from *A. actinomycetemcomitans* and *F. nucleatum* HtpG proteins: the C-terminus contains an extra inserted sequence (Table 11. bold in alignment). This insert appears to be exclusive to the Cytophaga-Flavobacterium-Bacteroides group HtpG proteins sequenced to date, and contains both a portion unique to each species (p18, underlined in alignment) and a conserved portion (p19). In contrast, the DnaK and GroEL of *P. gingivalis, A. actinomycetemcomitans,* and *F. nucleatum* align closely and display high homology. Examination of an alignment of Bacertoidacae HtpG molecules (Table 12) shows the insert can be roughly divided into 2 sections of about 30 amino acids each. The first-which contains p18-has relatively little sequence homology to the other species, only 6 of 36 (17%) amino acids are exact matches to the other Bacteroidacae HtpGs. The adjacent segment, p19, has 13 of 25 (52%) amino acids that are identical, similar to that between whole HtpG molecules from all 5 species (60-63%). BLAST analysis of p18 against the entire non-redundant protein database at GeneBank results in no hits except *P. gingivalis* HtpG. Similar searches using p19 produce significant hits with species in each of the Bacteroidacae genera (*B. fragilis* group; Non-*B. fragilis* group, Prevotella, Porphyromonas, Tanerella and “other”). Taken together we have concluded it is reasonable to assume that p18 is or contains an epitope that is unique to *P. gingivalis* HtpG and are currently investigating that hypothesis.

**Table 11:** Alignment of oral bacterial species HtpG amino acid sequences.

*A. actinomycetemcom.* HtpG	TPAVVSTDNDQMTTQMAKLFAAAG--QPVPEVKYTFELNPEHHLVKKVAEIAD-588
*F.nucleatum* HtpG	SASSLLAKGG-LSLEMEKTLSEMTNNNDMPKAEKVLAINPEHVLFNRLKSSVN 569
*P.gingivalis* HtpG	AILITQAEFMRRMRDMAQLQPGMSFYGELPDS-YNLVLNTDHPLIDRVLSGEK 582
*A. actinomycetemcom*. HtpG	IAD-------EAQFADWVELLLEQAMLAERGSLENP----------------- 614
*F.nucleatum* HtpG	SVN-------TEDFNKLVDVLYNQALLLEGFNIENP----------------- 595
*P.gingivalis* HtpG	GEKESVEPSLTELRAKIAELKAEEAKLLDEEKGKK*PEEIPVATKEAKENNAVE* 632
*A. actinomycetemcom*. HtpG	-----------------------------------------AAFIKRINKLLG 626
*F.nucleatum* HtpG	-----------------------------------------AEFIKNLNSLI 606
*P.gingivalis* HtpG	*QAKTEGSINDQLTK* YAQDNELIGQLIDLALLGSGLLTGEALAEFIRRSQRLL 684

Alignment of oral bacterial species HtpG amino acid sequences in the region of the 65 amino acid “Bacteroidacae insert” (Bold, underlined characters). The insert has not been described outside the Cytophaga, Flavobacteria and Bacteroides (CFB) group. Italic, bold–p18.

**Table 12:** Alignment of Bacteroidiacae HtpG C-terminal inserts

*B. thetaiotaomicron*	LKDSQKDKKEEDIPTAEKDELNELDKKWDELKNKKEGIFAG 643
*B. fragilis*	LKKKQEGKKDEDIPTAEKDELNDLDKKWDELKQQKDSIFAG 643
*P. intermedia*	LRQEQGKKKAEEITQEEKDDLKNTEESLSKQRTEKNDVIAN 646
*T. forsythia*	LREAQNKKKEDEITASEKEDLTNTNRKLTELRGQQNAILAE 647
*P. gingivalis*	LLDEEKGKKPEEIPVATKEAKENNAVEQAKTEGSINDQLTK 646
Consensus	* . : ** ::*. *: : . : . . : ::
*B. thetaiotaomicron*	YASNNKVIRQLIDLALLQNNMLKGEALNNFVKRSIELI- 681
*B. fragilis*	YAGKNKVVRQLIDLALLQNNMLKGEALNNFVKRSIELI- 681
*P. intermedia*	YAKGNNAIHQLIDLALLQNGMLKGAALDKFIKRSVDLIK 685
*T. forsythia*	YAAGNPIIGQLIDLALLGNGLLKGEALSRFIRRSVDLIR 686
*P. gingivalis*	YAQDNELIGQLIDLALLGSGLLTGEALAEFIRRSQRLL- 684
Consensus	** * : ******** ..:*.* ** .*::** *:

Alignment of 65 amino insert found in most CFB group bacteria, but not other bacterial groups. Bacteroidiacae HtpG C-terminal inserts shows p18 is much less conserved than p19. Underline/bold–p18 sequence. Dashed underline–p19 sequence. Amino acid identities across all species-^*^.

The mechanism that may connect anti-HtpG antibodies and progression of periodontitis is not known, but we speculate that it may well be that these antibodies block an interaction between HtpG and cellular receptors on macrophage [12]. Interactions between HtpG and the TLR4 and CD91 receptors induce the chemokine CXCL8, a chemoattractant for phagocytic cells in periodontitis [20], [21] and thus perpetuating the uncontrolled inflammation characteristic of periodontitis. Additionally, interactions between TLR4 and CD91 and HtpG induce inflammatory cytokines, including TNFα, as part of the innate immune response. Serum antibodies in 8 subjects with titers to *P. gingivalis* HtpG were found to reduce CXCL8 production in human monocytes in a dose dependent manner while serum antibodies from subjects without anti-HtpG activity did not [12].

The results reported here are similar to those of other chronic infections including *Heliobacter pylori*. World wide as much as 75% of the population has evidence of *H. pylori* infection but only subsets of these individuals manifest the peptic ulcers and stomach cancers associated with those chronic infections. The *H. pylori* chaperone hsp60 induces CXCL8 production in monocytic cells [22] and humoral immune response to a peptide epitope (pH9) of hsp60 is unique and seems to be associated with protection against *H. pylori* infection [23]. Intriguingly, when subjects with mucosa-associated lymphoid tissue lymphoma were examined for antibodies to the *H. pylori* hsp60 chaperone it was found that pre-treatment titers in patients whose tumors regressed after treatment were significantly higher than in patients whose tumors did not regress [24]. Low levels of antibodies to chaperones in disease subjects compared to controls has also been reported in inflammatory bowel disease [25], fungal infections [26] and other chronic bacterial infections [27].

The unique restriction of the *P. gingivalis* HtpG p18 peptide to the most important pathogen in periodontitis may have applications in vaccine and diagnostic arenas. The use of chaperones as vaccine candidates has been suggested by a number of investigators. Antibody to the Hsp90 homologue of *C. albicans* (Mycograb®, Novartis) has been shown to be effective in the treatment of disseminated fungal infections [28] in combination with amphotericin B. Other chaperones have been suggested as vaccine targets for diverse diseases in humans, cattle [29] and fish [30]. *P. gingivalis* GroEL has been used as a vaccine in a rat model of periodontitis that resulted in prevention of attachment loss [31], similar to that described here in humans. However, the authors cautioned that extensive homology between human and microbial chaperones may require use of peptides that do not induce cross-reactive antibodies with human hsp60 molecules. We believe this is the case with p18, which appears unique to *P. gingivalis* even to the exclusion of other Bacteroides species. The data presented here also suggests that serum antibodies to p18 may be useful in diagnosing periodontitis patients with extensive treatment requirements, identification of which would have substantial economic and epidemiological impact on the practice of periodontology.

## Materials and Methods

### Subjects

All work with human subjects was approved by the University of Michigan Institutional Review Board or the Queen's University, Belfast Ethics Committee, respectively. Each subject gave written individual informed consent and was advised that withdrawal from the study was available at their discretion at any time. The clinical condition of each subject was determined by examination of five clinical measurements: Probing pocket depth (PD) was determined to the nearest mm at six sites around each tooth and then averaged for all sites in each subject [32]. Bleeding on probing (BOP), was reported as a dichotomous measure and recorded as a percentage of the sites [33]. Clinical attachment loss (CAL) was determined at the same sites by measuring the distance between the cemento-enamel junction and the bottom of each pocket to the nearest mm and averaged [34]. The gingival index (GI) value represents the percentage of sites in each patient exhibiting redness associated with inflammation[35]. The plaque index value (PI) represents the percentage of sites found to have plaque biofilm accumulated at the gingival margin.

### Gingivitis Subjects

This group was examined from archived samples described in a previous report and was included here to address the potential differences between response to *P. gingivalis* HtpG and human Hsp90. This retrospective study focused on a population of individuals living in southwestern Michigan [15], [16] with gingivitis, a form of periodontal disease with no or minimal tissue destruction that is reversible with active oral hygiene. Subjects were recruited into the original study based on their membership in the rural community, not their oral health status, and all essentially presented with gingivitis or mild periodontitis. The clinical data and archived serum samples collected from 50 subjects were used. The average age was 31.6 years (range: 18–66) and 58% were female. Other characteristics of these subjects are found in Table 1.

### Chronic Periodontitis Subjects

Subjects were recruited at the Michigan Center for Oral Health Research. Subject inclusion was based on: possession of at least 20 teeth, no periodontal treatment or antibiotic-related therapy for medical or dental reasons for 3 months before study inclusion, no history of long-term treatment with medications known to affect periodontal status, and no history of metabolic bone diseases. Healthy control subjects (n = 49) were recruited who had less than 3 mm of CAL, no pocket depth (PD)s greater than 4 mm, no radiographic bone loss, and less than 20 sites with bleeding on probing. Chronic periodontitis (CP) subjects (n = 50) exhibited at least 4 sites with evidence of radiographic bone loss, mean CAL >3mm, PD >4 mm and bleeding on probing (Table 1).

### Aggressive Periodontitis Subjects

Studies were done with serum samples from an ongoing study of Aggressive Periodontitis (AP) subjects (n = 24) and age matched control subjects (n = 21) living in Northern Ireland. AP subjects included in this investigation were 30.3±4.0 years of age at the time of clinical and radiographic examination, diagnosed with severe periodontitis, and had a minimum of 4 sites with a probing depth of at least 5 mm and CAL loss of at least 2 mm. Age matched controls without periodontitis were recruited from regular attendees at the Queen's University School of Dentistry. Colonization of AP subjects by *P. gingivalis* was determined by PCR using primers specific for the *P. gingivalis* 16S rRNA gene [17].

### Bacterial strains and culture conditions

*Porphyromonas gingivalis* (ATCC 33277) and *Fusobacterium nucleatum* (ATCC 25586) were obtained from the American Type Culture Collection. *Porphyromonas gingivalis* strain W83 was a gift from Dr. Donald Clewell, University of Michigan School of Dentistry. The bacteria were maintained by weekly transfer in an anaerobe chamber (Coy Manufacturing, Grass Lake, MI) at 37°C on PRAS Brucella agar plates (Anaerobe Systems, Morgan Hill, CA) in a 5% hydrogen, 10% carbon dioxide, 85% nitrogen atmosphere. Broth cultures were grown in a mixture of 50% Brain Heart Infusion Broth, 50% Trypticase Soy Broth and 5 gram/L Yeast Extract supplemented with 0.01 gm/L Sodium Bisulfite, 5 mg/L hemin and 5ug/L Vitamin K.

### Antigen preparation

#### Cloning and purification of *P. gingivalis* and *F. nucleatum* rHtpG

The full length sequence of *P. gingivalis* HtpG (GenBank ascension number AF176245) and *F. nucleatum* HtpG (GenBank ascension number EDK88176) were obtained from the National Center for Biotechnology Information (www,ncbi.nlm.nih.gov). PCR primers were designed to produce full-length products that were subsequently inserted into pCR®T7 TOPO® cloning vector following the manufacturer's instructions (Invitrogen, Carlsbad, CA.). Clones of One Shot® Chemically Competent *E. coli* (Invitrogen) transformed with the vector were ampicillin selected and then screened by PCR. Inserts that produced amplicons of the correct size were sequenced to verify the full-length insert (Biomedical Research Core Facilities, University of Michigan, Ann Arbor, MI). Plasmids with in-frame inserts were used to transform TOP10F BL21(DE3)pLysS *E.coli* (Invitrogen) cells that were subsequently induced with 100 µM isopropyl β-D-1-thiogalactopyranoside for 4 hours to produce a fusion protein with 6 consecutive histidine (6xhis) residues preceding the N-terminal of the cloned proteins. The protein was purified to >95%, as determined by SDS-PAGE electrophoresis, by Ni-agarose chromatography (Ni-NTA™ Agarose, Qiagen, Valencia, CA).

#### Preparation of HtpG peptide (p18)

A 36 amino aid peptide (KKPEEIPVATKEAKENNAVEQAKTEGSINDQLTKYA) was synthesized on an Applied Biosystem 433A peptide synthesizer at the University of Michigan department of Chemistry using Fmoc amino acids from AnaSpec (San Jose, CA). The crude peptide was purified by HPLC and the purity (∼98%) confirmed by MALDI-TOF analysis.

### ELISA assays

All assays were carried out in 384-well microtiter plates (NUNC™ black MaxiSorp, Rochester, NY) using 4-methyumbliferone phosphate as a substrate for alkaline phosphatase-labeled tracers. Antigens were coated onto the plates in 25 µl/well volumes in sodium carbonate/bicarbonate buffer (0.05M, pH 9.5) and incubated overnight at 4°C. Plates were washed 3 times with 0.02 M phosphate buffered saline (PBS, pH 7.4) and PBS with 1% bovine serum albumin (PBS-BSA) added to block unoccupied protein binding sites (100 µL/well). After an additional hour of incubation at room temperature plates were washed with PBS with 0.125% NP40 three times and human serum (or control rabbit serum) diluted 1:100 in PBS-BSA (25 uL/well) added to the plates in triplicate and incubated overnight at 4°C. Plates were then washed 3 times with PBS with 0.125% NP40 and detection reagents added as described below. Blocked wells not coated with antigen were used as negative controls for each individual serum sample. Data is expressed as net relative florescent units (RFU) calculated by subtracting the average of 3 control wells from the average of 3 antigen coated wells and were repeated at least 3 times each.

### Antibodies to *P. gingivalis* Whole Cell Lysate

*P. gingivalis* cells (strain W83) were grown to mid-log phase, washed by centrifugation at 10,000×g 3 times with sterile PBS and resuspended at an OD_600_ of 1.0 in water. The cells were sonicated for 2 minutes on ice three times and centrifuged at 10,000×g for 20 minutes. Total protein was determined and the clarified lysate diluted to 10 µg/mL for coating. Serum IgG binding was determined as described above using alkaline phophatase-labeled anti-human IgG(γ) antibodies and 4-methylubelliferyl phosphate (1 µg/mL in 0.2 M TRIS, pH 9.5) (Sigma, St. Louis).

### Antibodies to *P. gingivalis* HtpG and *F. nucleatum* HtpG

Recombinant HtpG proteins were dissolved in carbonate buffer to 1 µg/mL for plate coating. Total IgG(γ) binding was determined using the same second antibodies as described for the whole cell lysate assay above.

### Antibodies to *P. gingivalis* HtpG peptide 18

The peptide was dissolved in carbonate buffer to 10 µg/mL for plate coating. Total serum IgG(γ) binding was determined using the same second antibodies as above.

### Detection of *P. gingivalis* and *F. nucleatum* colonization in plaque samples

The detection of *P. gingivalis* in pooled plaque samples from the gingivitis subjects was done using a slot immunoblotting method as described previously [36]. Colonization of plaque samples collected simultaneously with the serum in the CP groups were evaluated by real-time PCR as described [37] using primers specific for *P. gingivalis* (forward:5′-CATAGATATCACG-AGGAACTCCGATT-3′; reverse 5′-AAACTGTTAGCAACTACCGATGTGG-3′) and *F. nucleatum* (forward: 5′-AAATATGTTGAATTCTGGAAAGAGTTTG-3′; reverse: 5′-TGAACTCCAGCTTTTATACTTCTACCAA-3′). Percentage of the total flora for each species was calculated by dividing the number of target organisms by the total number of bacteria as determined by realtime PCR using 16S rRNA primers that reacted with all bacterial species (forward: 5′-CCATGAAGTCGGAATCGCTAG-3′; reverse: 5′-GCTTGACGGGCGGTGT-3′). The presence of *P. gingivalis* in plaque from the AP subjects was determined by PCR as described earlier [17] using the same primers.

### Statistical Analysis of anti-HtpG levels

Statistical computations were done using STATISTICA™ v. 6.0 (StatSoft, Omaha, NE). Comparison of two means was performed using *t*-tests. The relationships between specific indices of periodontal disease, antibody levels, and colonization were assessed by ANOVA. Fisher's method for multiple comparisons was used for group comparisons. Log transformation of data was performed where appropriate. Results with p-values of ≤ 0.05 were considered significant.

## Supporting Information

Table S2 Antibody to *P. gingivalis* HtpG and human Hsp90 serum levels in CP subjects.
(0.03 MB DOC)

Table S1 Correlation of human Hsp90 levels to Anti-HtpG levels in 95 CP patients and healthy controls.
(0.03 MB DOC)

## References

[1] Socransky SS, Haffajee AD (1997). The nature of periodontal diseases.. AnnPeriodontol.

[2] Socransky SS, Haffajee AD, Cugini MA, Smith C, Kent RL (1998). Microbial complexes in subgingival plaque.. JClinPeriodontol.

[3] Progulske-Fox A, Kozarov E, Dorn B, Dunn W, Burks J (1999). Porphyromonas gingivalis virulence factors and invasion of cells of the cardiovascular system.. J Periodontal Res.

[4] Kozarov E, Sweier D, Shelburne C, Progulske-Fox A, Lopatin D (2006). Detection of bacterial DNA in atheromatous plaques by quantitative PCR.. Microbes Infect.

[5] Goulhen F, Grenier D, Mayrand D (2003). Oral microbial heat-shock proteins and their potential contributions to infections.. Crit Rev Oral Biol Med.

[6] Prohaszka Z, Fust G (2004). Immunological aspects of heat-shock proteins-the optimum stress of life.. Mol Immunol.

[7] Shamaei-Tousi A, D'Aiuto F, Nibali L, Steptoe A, Coates AR (2007). Differential regulation of circulating levels of molecular chaperones in patients undergoing treatment for periodontal disease.. PLoS ONE.

[8] Young D, Lathigra R, Hendrix R, Sweetser D, Young RA (1988). Stress proteins are immune targets in leprosy and tuberculosis.. ProcNatlAcadSciUSA.

[9] Lopatin DE, Shelburne CE, Van Poperin N, Kowalski CJ, Bagramian RA (1999). Humoral immunity to stress proteins and periodontal disease.. J Periodontol.

[10] Rams TE, Listgarten MA, Slots J (2006). Actinobacillus actinomycetemcomitans and Porphyromonas gingivalis subgingival presence, species-specific serum immunoglobulin G antibody levels, and periodontitis disease recurrence.. J Periodontal Res.

[11] Yamazaki K, Ueki-Maruayama K, Honda T, Nakajima T, Seymour GJ (2004). Effect of periodontal treatment on the serum antibody levels to heat shock proteins.. Clin Exp Immunol.

[12] Shelburne CE, Coopamah MD, Sweier DG, An FY, Lopatin DE (2007). HtpG, the Porphyromonas gingivalis HSP-90 homologue, induces the chemokine CXCL8 in human monocytic and microvascular vein endothelial cells.. Cell Microbiol.

[13] Luoma J, Hiltunen T, Sarkioja T, Moestrup SK, Gliemann J (1994). Expression of alpha 2-macroglobulin receptor/low density lipoprotein receptor-related protein and scavenger receptor in human atherosclerotic lesions.. J Clin Invest.

[14] Kinane DF, Mooney J, Ebersole JL (1999). Humoral immune response to Actinobacillus actinomycetemcomitans and Porphyromonas gingivalis in periodontal disease.. Periodontol 2000.

[15] Bagramian RA, Farghaly MM, Lopatin D, Sowers M, Syed SA (1993). Periodontal disease in an Amish population.. JClinPeriodontol.

[16] Bagramian RA, Farghaly MM, Lopatin D, Sowers M, Syed SA (1994). A comparison of periodontal disease among rural Amish and non-Amish adults.. JClinPeriodontol.

[17] Mullally BH, Dace B, Shelburne CE, Wolff LF, Coulter WA (2000). Prevalence of periodontal pathogens in localized and generalized forms of early-onset periodontitis.. JPeriodontal Res.

[18] Sweier DG, Shelburne CE, Cameron J, Lopatin DE (2004). Localizing antibody-defined immunoreactivity in Porphyromonas gingivalis HtpG recognized by human serum utilizing selective protein expression.. J Immunol Methods.

[19] Chatterjee A, Dimitropoulou C, Drakopanayiotakis F, Antonova G, Snead C (2007). Heat shock protein 90 inhibitors prolong survival, attenuate inflammation, and reduce lung injury in murine sepsis.. Am J Respir Crit Care Med.

[20] Hosokawa Y, Hosokawa I, Ozaki K, Nakae H, Matsuo T (2006). Proinflammatory effects of tumour necrosis factor-like weak inducer of apoptosis (TWEAK) on human gingival fibroblasts.. Clin Exp Immunol.

[21] Okada H, Murakami S (1998). Cytokine expression in periodontal health and disease.. Crit Rev Oral Biol Med.

[22] Lin SN, Ayada K, Zhao Y, Yokota K, Takenaka R (2005). Helicobacter pylori heat-shock protein 60 induces production of the pro-inflammatory cytokine IL8 in monocytic cells.. J Med Microbiol.

[23] Yamaguchi H, Osaki T, Kai M, Taguchi H, Kamiya S (2000). Immune response against a cross-reactive epitope on the heat shock protein 60 homologue of Helicobacter pylori.. InfectImmun.

[24] Takenaka R, Yokota K, Mizuno M, Okada H, Toyokawa T (2004). Serum antibodies to Helicobacter pylori and its heat-shock protein 60 correlate with the response of gastric mucosa-associated lymphoid tissue lymphoma to eradication of H. pylori.. Helicobacter.

[25] Huszti Z, Bene L, Kovacs A, Fekete B, Fust G (2004). Low levels of antibodies against E. coli and mycobacterial 65kDa heat shock proteins in patients with inflammatory bowel disease.. Inflamm Res.

[26] Matthews RC, Burnie JP, Howat D, Rowland T, Walton F (1991). Autoantibody to heat-shock protein 90 can mediate protection against systemic candidosis.. Immunology.

[27] Zugel U, Kaufmann SH (1999). Role of heat shock proteins in protection from and pathogenesis of infectious diseases.. ClinMicrobiolRev.

[28] Burnie JP, Carter TL, Hodgetts SJ, Matthews RC (2006). Fungal heat-shock proteins in human disease.. FEMS Microbiol Rev.

[29] Koets A, Hoek A, Langelaar M, Overdijk M, Santema W (2006). Mycobacterial 70 kD heat-shock protein is an effective subunit vaccine against bovine paratuberculosis.. Vaccine.

[30] Sudheesh PS, LaFrentz BR, Call DR, Siems WF, LaPatra SE (2007). Identification of potential vaccine target antigens by immunoproteomic analysis of a virulent and a non-virulent strain of the fish pathogen Flavobacterium psychrophilum.. Dis Aquat Organ.

[31] Lee JY, Yi NN, Kim US, Choi JS, Kim SJ (2006). Porphyromonas gingivalis heat shock protein vaccine reduces the alveolar bone loss induced by multiple periodontopathogenic bacteria.. J Periodontal Res.

[32] Loe H, Theilade E, Jensen SB (1965). Experimental gingivitis man.. JPeriodontol.

[33] Offenbacher S (2005). Commentary: clinical implications of periodontal disease assessments using probing depth and bleeding on probing to measure the status of the periodontal-biofilm interface.. J Int Acad Periodontol.

[34] Wolff LF, Liljemark WF, Pihlstrom BL, Schaffer EM, Aeppli DM (1988). Dark-pigmented Bacteroides species in subgingival plaque of adult patients on a rigorous recall program.. J Periodontal Res.

[35] Silness J, Loe H (1966). Periodontal disease in pregnancy. 3. Response to local treatment.. Acta OdontolScand.

[36] Van Poperin N, Lopatin DE (1991). Slot immunoblot assay for detection and quantitation of periodontal disease-associated microorganisms in dental plaque.. JClinMicrobiol.

[37] Shelburne CE, Prabhu A, Gleason RM, Mullally BH, Coulter WA (2000). Quantitation of Bacteroides forsythus in subgingival plaque comparison of immunoassay and quantitative polymerase chain reaction.. J Microbiol Methods.

