# Supplementary material for: Serum Antibodies to Porphyromonas gingivalis Chaperone HtpG Predict Health in Periodontitis Susceptible Patients
Source: PLoS One. 2008 Apr 23;3(4):e1984. doi: 10.1371/journal.pone.0001984 (PMC2291562; doi:10.1371/journal.pone.0001984)
Supplement: Table S2 — Antibody to P. gingivalis HtpG and human Hsp90 serum levels in CP subjects. (0.03 MB DOC) [file pone.0001984.s001.doc]

**Table S2. Antibody to P. gingivalis HtpG and human Hsp90 serum** levels in CP subjects.

|  | Healthy | Severe Periodontitis | p-value (*t*-test) |
| --- | --- | --- | --- |
| Mean Serum Anti-HtpG (ELISA Units) | 13,395 ± 4814 | 7,877 ± 3906 | 0.016 |
| Mean Serum Hsp90 (ng/mL) | 13.4 ± 4.8 | 12.3 ± 6.6 | NS |

NS – not significant

Serum Hsp90 levels were determined using an Hsp90α ELISA Assay Kit (Catalog Number: EKS-895, Assay Designs, Ann Arbor, Michigan) according to the manufacturer’s instruction. Antibody levels were measured as described in the text.
