# Supplementary material for: Serum Antibodies to Porphyromonas gingivalis Chaperone HtpG Predict Health in Periodontitis Susceptible Patients
Source: PLoS One. 2008 Apr 23;3(4):e1984. doi: 10.1371/journal.pone.0001984 (PMC2291562; doi:10.1371/journal.pone.0001984)
Supplement: Table S1 — Correlation of human Hsp90 levels to Anti-HtpG levels in 95 CP patients and healthy controls. (0.03 MB DOC) [file pone.0001984.s002.doc]

**Table S1. Correlation of human Hsp90 levels to Anti-HtpG levels in 95 CP patients and healthy controls.**

|  | R | p-value |
| --- | --- | --- |
| Anti-*P. gingivalis* HtpG | 0.52 | NS |
| Anti-*F. nucleatum* HtpG | -0.05 | NS |
| Anti-*P. gingivalis* HtpG p18 | -0.17 | NS |

NS-not significant.

Serum Hsp90 levels were determined using an Hsp90α ELISA Assay Kit (Catalog Number: EKS-895, Assay Designs, Ann Arbor, Michigan) according to the manufacturer’s instruction. Antibody levels were determined as described in the text.
